# Decolouring. The Racial Imprints of Upward Mobility in Lima's Dominant Class

**DOI:** 10.1111/1468-4446.70023

**Published:** 2025-08-03

**Authors:** Mauricio Rentería

**Affiliations:** ^1^ The University of Manchester Manchester UK

**Keywords:** class, Lima, race, upward mobility, whitening

## Abstract

A significant body of literature highlights the fluid and adaptable nature of racial categories in Latin America, often invoking the concept of ‘whitening’ to explain how upwardly mobile individuals reshape their racial or ethnic identities by adopting cultural and social traits associated with class privilege and ‘whiteness’. This study builds on these discussions. Drawing on 42 interviews, it examines the racial imprints of class mobility within Lima's dominant class, focussing particularly on ‘*Mestizos’* in the sample. I show that upward mobility has distinct racialised effects for this group, especially when contrasted with the experiences of their ‘Afro‐Peruvian’ counterparts. Whereas for the latter, upward social mobility engenders little change to their racial status, for ‘*Mestizos’*, it involves shedding the stigmatised racial label ‘*Cholo’*. Rather than achieving a symbolically higher racial status, for ‘*Mestizos’* mobility prompts a process I term ‘decolouring’, characterised by distancing from racial stigma and navigating a heightened sense of racial ambiguity.

## 1 Introduction

In Peru, as in most Latin American countries, class divisions have traditionally been closely tied to race, associating whiteness with material and cultural power, while linking mixed‐race and indigenous populations with poverty and cultural inferiority (Bourricaud 1970; De la Cadena and et al. 1995; De la Cadena 1998; Fuenzalida 1970). These racialised class dynamics are most evident in the concept of ‘whitening’ as a perceived outcome of upward social mobility. Rather than viewing racial identity as fixed, a significant body of scholarship demonstrates how individuals alter their racial and ethnic identities by attaining higher social status. This transformation often entails abandoning customs and practices associated with Indigenous culture and adopting elements of an urban and ‘modern’ lifestyle, often facilitated by migration to cities. Scholars have described this process as ‘de‐Indianisation,’ referring to the shift from ‘*Indio’* to ‘*Mestizo’* (De la Cadena and et al. 1995; De la Cadena 1998), and as ‘*cholificación*,’ where individuals become identified as ‘*Cholos’* (Bourricaud 1970; Quijano 1969). However, apart from historical research (Cosamalón 2017), most of this literature has concentrated on the lower levels of Peru's class structure, leaving key questions unanswered about how mobility into the upper echelons of society influences individuals' racial identities. This raises critical questions: Can we identify an equivalent process of ‘ethnic mobility’ (De la Cadena and et al. 1995, 333) occurring when non‐White individuals ascend into elite social spaces? Furthermore, can such a process be understood as a form of ‘whitening’?

In this article, I explore how social mobility shapes the racial status of upwardly mobile Limeños, particularly those who self‐identify as ‘*Mestizos’*. Drawing on testimonies from 42 members of Lima's dominant class—32 upwardly mobile and 10 intergenerationally stable—I argue that the racial implications of mobility for ‘*Mestizo’* individuals extend beyond discussions focussed on ‘whitening’ (Kogan and Galarza 2017; Mitchell 2017; Vargas 2015; Viveros Vigoya 2015; Wade 1993). By analysing how they identify themselves and their relatives in both class and racial terms, I demonstrate that mobility produces different outcomes for ‘Afro Peruvian’ and ‘*Mestizo’* individuals in the sample. For ‘Afro Peruvian’ individuals, achieving higher social status has a limited impact on their racial identity. The challenges they face in integrating into middle‐ and upper‐class settings are mostly framed as class‐related, with little influence on their racial positioning. In contrast, ‘*Mestizo’* individuals report that upward mobility enables them to distance themselves from the stigmatized status of ‘*Cholos’*, a derogatory term equivalent to ‘*Mestizo’*. To capture the complexities of the racialised experiences of ‘*Mestizo’* individuals, I introduce the concept of ‘decolouring’. This notion highlights how ‘*Mestizo’* individuals distance themselves from a stigmatised racial identity without acquiring a symbolically higher racial identification. As a result, they experience heightened racial ambiguity and a continuous risk of encountering racism, particularly in elite, ‘White’‐dominated environments.

## 2 Malleable and Rigid Racial Boundaries

### 2.1 The Logics of Whitening

In Latin America, the fluidity of racial categories is best represented by the idea of ‘whitening’. Essentially, ‘whitening’ involves a process by which non‐White individuals assimilate to the values and norms of ‘whiteness’—whether this is done purposely or unintentionally (Wade 1993). Whiteness encompasses ideologised images of privilege, including evaluations of appearance and behaviour (Viveros Vigoya 2015) that serve as an unmarkerd norm (Frankenberg 1997). The scholarship identifies different forms of whitening, from interracial marriage to geographical mobility and integration into symbolically ‘white’ spaces (Telles and Esteve 2019; Vasquez 2014; Wade 1993).

The most prominent and widely studied form of whitening is tied to upward social mobility. This process involves attaining higher status through professional achievements or educational qualifications, adopting changes in lifestyle and personal appearance, and integrating into elite circles dominated by ‘White’ individuals from privileged backgrounds. In Peru and other Latin American countries, this form of whitening has traditionally functioned as a ‘utopia’, promising beauty and socioeconomic advancement to non‐White populations striving to become ‘White’ (Portocarrero 2013).

Historical research has uncovered instances of non‐white Limeños attaining ‘white’ status through upward social mobility (Cosamalón 2017). In this sense, whitening can be understood as a symbolic shift towards a social status associated with ‘whiteness’. This may involve individuals who, by virtue of their elevated social position, are able to ‘pass’ as white (Brubaker 2016; Hobbs 2014), acquire a racial status closer to whiteness—similar to the ‘*mulatto* escape hatch’ in Brazil (Degler 1971)—or be treated as white without being fully incorporated into the category, as suggested by Bonilla‐Silva's (2002) ‘honorary white’ thesis for groups like light‐skinned Latinos in the United States.

While the concept of ‘whitening’ often emphasises acquiring a symbolically higher racial status through mobility, some scholars focus on its role in shedding a symbolically ‘inferior’ racial identity. Wade's (1993) research in Colombia illustrates this, showing that whitening, achieved through social and geographical mobility, is more about distancing oneself from ‘Blackness’ than fully attaining ‘White’ status. A similar process of detachment from a stigmatised racial identity emerges in the literature on ‘de‐Indianisation’ in rural Peru. De la Cadena and et al. (1995), De la Cadena (1998) illustrates how ‘*Indios’* become ‘*Mestizos’* by migrating to urban areas and adopting an urban lifestyle. Rather than simply a process of assimilation, this transformation often involves a form of resistance to the dominant group's essentialist representations of ‘*Indios’*—for example, the notion that to be considered an ‘*Indio’*, one must lack formal education. In such cases, ethnoracial identity and class intersect in what De la Cadena (1995: 333) calls ‘ethnic mobility’, involving occupational and lifestyle changes alongside practices like speaking Spanish instead of Quechua and adopting urban dress.

What enables the possibility of ‘ethnic mobility’ or the ‘de‐Indianisation’ of individuals is the prevalence of the ‘*Mestizaje’* ideology in Peru. However, for elites, ‘*Mestizaje’* also represented an effort to assimilate ‘Black’ and ‘Indigenous’ people into a nation that was seen as ‘moving towards whiteness’ (Wade 2008, 181). As in other countries where the ideology of ‘*Mestizaje’* gained prominence, deeply racialised practices persisted beneath the universal rhetoric that celebrated notions of ‘*Mestizo’* individuals (Mitchell 2017; Moreno Figueroa 2022). These practices elevated ‘whiteness’ in its various forms while rejecting cultural products and practices associated with non‐White identities—whether markers of Indigeneity, Blackness, or mixed‐race heritage.

In Peru, the imagery surrounding the category of ‘*Cholo’* offers a striking example of how racism intersects with the idea of ‘*Mestizaje’*. Although often used interchangeably with ‘*Mestizo’*, the term ‘*Cholo’* carries more contested and conflict‐ridden meanings. While ‘*Mestizo’* typically functions as a neutral and sometimes vague term for racial mixture, ‘*Cholo’* evokes sharper, more polarising connotations. Initially, the figure of the ‘*Cholo’* was celebrated by some as a symbol of cultural blending between ‘Indigenous’ and ‘Western’ traditions (Bourricaud 1970, 1975; Quijano 1969; Varallanos 1962). However, in major cities like Lima, the term evolved into a derogatory synonym for ‘*Mestizo’*. Its everyday usage now mostly encapsulates negative stereotypes, functioning as a slur that conveys associations with deprivation and racial inferiority. Far from being monopolised by the most privileged ‘White’ sectors of Peruvian society, ‘*choleo’*—the act of using ‘Cholo’ as an insult—has been deployed across all levels of society, from top to bottom (Avilés 2018; Nugent 2021a, 2021b). This widespread use reflects both the inherent instability of the ‘*Cholo’* category and the broad recognition of its negative connotations.

### 2.2 Enforcing Racial Boundaries

If the possibilities associated with ‘whitening’ highlight the cultural malleability of racial dynamics, the prevalence of what some describe as ‘colourism’ underscores the enduring significance of physical features in maintaining racial boundaries in Latin America (Dixon 2019; Hunter 2007; Telles 2014). Colourism refers to the stigmatisation and discrimination of individuals based on their skin colour and other racialised physical traits.

Golash‐Boza's (2010) research on an Afro‐Peruvian community illustrates the persistence of colourism among Black Peruvians who cannot ‘whiten’ due to the fixed nature of ‘Blackness’ as a racial category. She stresses the importance of distinguishing between race and colour labels conceptually. Race refers to mutually exclusive groups of people defined by shared physical and cultural traits, as seen in the Afro‐Peruvian population, who identify with an imagined collective experience of ‘Blackness’. In contrast, colour encompasses a spectrum of skin tones that do not conform to mutually exclusive categories. Racial categories, whether institutionalised by the state or used in everyday interactions, tend to construct group‐based identities, whereas colour labels do not.

While concepts like ‘whitening’ and ‘de‐Indianisation’ highlight the fluidity of racialisation processes, the prevalence of ‘colourism’ underscores the limits of these processes. However, these frameworks offer only a partial view of the racialisation experiences of upwardly mobile ‘*Mestizos’* in the upper echelons of Lima. To fully understand the complexities of how mobility influences their racial positioning, I propose the concept of ‘decolouring’. As I will demonstrate in the following sections, this notion involves distancing oneself from a stigmatised racial status, akin to ‘de‐Indianisation’, but without fully assimilating into a ‘White’ identity due to the ongoing influence of colourism.

## 3 Methodology

In this study, I focus on the experiences of individuals who reach the upper levels of Lima's class structure. Following recent adaptations of Bourdieu's (2010) class perspective, I focus on individuals within the ‘dominant class’ (W. Atkinson 2017; Hansen et al. 2009; Rentería and Zárate 2022) and adopted a qualitative analysis based on in‐depth interviews. I began my fieldwork in September 2022 and completed it in December 2022. During this period, I recruited participants and conducted a total of 42 interviews.

To recruit participants, I based my inclusion criteria on occupation and age. For occupation, I included individuals working in roles classified within the ‘dominant class’ according to Rentería and Zárate's (2022) recent Bordieuan‐inspired class scheme for Peru. This class gathers around 9% of Lima's population (4% in Peru), with occupations associated with high levels of economic^1^ and cultural capital (measured by education level) across various fields, ranging from managerial and executive positions in the private sector to architects, lawyers, and university professors. I selected participants from culturally dominant occupations (16, with 8 female and 8 male), such as artists and university professors; those with balanced economic and cultural capital (7, with 4 female and 3 male), such as physicians and lawyers; and those from economically dominant occupations (19, with 5 female and 14 male), including CEOs and financial analysts. Nearly all (40 out of 42) participants attended the most prestigious public and private universities in the country and the majority resided in some of the most affluent neighbourhoods in Lima (33 out of 40 currently residing in Lima).

I focussed on respondents aged 25 or older, excluding younger individuals due to their limited professional experience and career instability. I assumed that those aged 25 and above would have more established career prospects compared to younger participants, who had either recently graduated or were in their first jobs. The sample includes 10 respondents aged 25–35, 18 aged 36–49, and 14 aged 50–66.

Participants were contacted through LinkedIn invitations, followed by snowball sampling initiated via these initial contacts. I chose this approach because LinkedIn is widely used by individuals in prestigious occupations and had previously proven effective for reaching high‐level professionals in studies of the Peruvian upper class (Rentería et al. 2020).

This study categorised participants according to their mobility status, using the widely accepted criterion of their parents' occupation when the participants were 14 years old. Given that the focus is primarily on the experiences of upwardly mobile individuals, I intentionally included more participants who graduated from public universities and schools, assuming that these institutions disproportionately attract individuals from underprivileged backgrounds. However, each respondent's mobility was attributed according to their description of their parents' occupations during their childhood. Respondents were grouped into three categories based on their mobility status: Established, Short‐range, or Long‐range. The first group consisted of 10 respondents whose parents held occupations within the dominant class,^2^ the second included 20 respondents whose parents worked in mid‐level positions within the ‘intermediate class’, such as secretaries, teachers, or police officers, and the third group comprised 12 respondents whose parents held jobs typically associated with the ‘working class’, such as grocers, merchants, or security guards. The suitability of this approach is evident when comparing the educational backgrounds of respondents' ancestors: university attendance was significantly higher among the Established than the upwardly mobile—fathers (9/10 vs. 10/28), mothers (4/10 vs. 5/29), paternal grandfathers (4/6 vs. 4/28), and maternal grandfathers (4/6 vs. 2/21).^3^

It is important to note that this occupation‐based criterion does not fully capture the diversity within both the Established and upwardly mobile groups—for instance, differences between families with histories tied to certain high‐status economic or culturally dominant occupations. Nonetheless, as the discussion will show, this classification proves useful for distinguishing between relatively similar and divergent class trajectory experiences.

I did not include racial identity as part of the recruitment status. Instead, I allowed respondents to identify themselves during conversations, either through informants' own descriptions of their experiences or through my direct questioning towards the end of the interview. This meant that although most LinkedIn profiles included photos, I did not use these as a proxy for respondents' racial identification.^4^

I employed a qualitative approach, focussing on respondents' experiences and understandings of their social trajectories and racialisation. This aligns with established qualitative traditions that view social mobility as a deeply meaningful, emotional lived experience (Friedman 2014; Mallman 2017; Reay 2017). I conducted semi‐structured life history interviews, centred on respondents' voices and how they construct and reconstruct their life stories (R. Atkinson and et al. 2012). This method suited my focus on social mobility by highlighting narratives of social processes across individuals' lives.

Following Becker and Harrison (2008), life history interviewing allowed me to examine the ‘formation of the individual’, where behaviour is continuously reshaped through symbolically mediated processes influenced by others' expectations. Like other interview techniques, it provided insight into respondents' current emotions and perspectives, often leading them to interpret their past in ways that reflect their present self‐view and aspirations for the future—how they wished to position themselves in their ongoing life narrative, rather than simply recounting past events (R. Atkinson and et al. 2012, 120; Block 2017, 35).

All interviews were conducted in Spanish and recorded with the respondents' written consent. I analysed the interviews using thematic analysis of the narrative accounts respondents provided about their trajectories. I began the analysis during fieldwork, recording basic information about each interviewee and the setting, along with my initial reflections on the conversation, in a notebook after each interview. Once fieldwork was complete, I transcribed each interview in Spanish, ensuring that the transcripts captured the respondents' speech patterns, including inflexions, silences, and pauses. Written informed consent was obtained from all participants, and I have pseudonymised all names in the article.

## 4 Findings

### 4.1 Downplaying Class Privilege

Table 1 summarises respondents' responses to questions concerning class and racial identification of themselves and their relatives. Concerning social class, most interviewees found it difficult to categorise themselves within a specific stratum. This resonates with discussions about people's hesitation when asked about their ‘class identities’ (Bottero 2004; Payne and Grew 2005; Reay 1998; Savage et al. 2001; Skeggs 1997). In particular, these responses manifest an avoidance of most respondents in identifying themselves with class privilege. This was evidenced in different ways. For example, the minority of interviewees who positioned themselves in the upper echelons of the class structure used categories that diverged from traditional class terminology. These categories, such as socioeconomic status and income groups, are perceived as more descriptive or morally neutral.

**TABLE 1:** Class self‐identification, racial self‐identification, and racial identification of parents by mobility status.

Class self‐identification	Racial self‐identification	Race mother	Race father
Established			
A	Don't know	White	White
Middle class	Don't know	White	White
Upper middle class	Mestizo	White	White
A‐	Mestizo	White	White
Privileged	Mestizo	White	White
No response	Mestizo	White	White
No response	White	White	Mestizo
A	Mestizo	Mestizo	Mestizo
*Missing*	*Missing*	*Missing*	*Missing*
*Missing*	*Missing*	*Missing*	*Missing*
Long‐range			
Middle class	Afro‐peruvian^a^	Afro‐peruvian	N/A
Middle class	Afro‐peruvian	*Missing*	*Missing*
No response	Afro‐peruvian	Mestizo	Afro‐peruvian
Middle class	Mestizo	Mestizo	Mestizo
Middle class	Mestizo	Mestizo	Mestizo
High income	Mestizo	White	Mestizo
Upper middle class	Mestizo	Mestizo	Mestizo
A	Mestizo	Mestizo	Mestizo
A	Mestizo	Mestizo	Mestizo
A	Mestizo	Mestizo	Mestizo
Upper middle class	Mestizo	Mestizo	White
Middle class	Mestizo	Mestizo	Mestizo
B	Mestizo	Mestizo	Mestizo
No response	Mestizo	Mestizo	White
Middle class	Mestizo	Mestizo	Mestizo
Upper middle class	Mestizo	Mestizo	Afro‐peruvian
Top quintile	White	White	White
Short‐range			
A‐	Mestizo	Mestizo	Mestizo
Upper middle class	Mestizo	Mestizo	White
A2	Mestizo	*Missing*	*Missing*
No response	Mestizo	Mestizo	Mestizo
No response	Mestizo	Mestizo	Mestizo
B	Mestizo	Mestizo	Mestizo
Upper middle class	Mestizo	Mestizo	Mestizo
Middle class	Mestizo	Mestizo	Mestizo
Middle class	Mestizo	Mestizo	Mestizo
No response	Mestizo	Mestizo	Mestizo
*Missing*	Mestizo	*Missing*	*Missing*
Upper middle class	Mestizo	*Missing*	*Missing*
No response	Mestizo	Mestizo	Mestizo
Middle class	Mestizo	White	Mestizo
*Missing*	Afro‐peruvian	*Missing*	*Missing*

^a^ ‘Afro‐Peruvian’ includes those who responded ‘Afro‐Peruvian’ and ‘Black’.

Underlying these responses is a widespread negative depiction of the dominant sectors in Peru. The empirical data consistently revealed negative associations whenever the term ‘upper class’ was mentioned. Most Established and upwardly mobile respondents shared similar negative perceptions of the upper class, with comparable remarks distancing their behaviours and values from those attributed to this privileged sector of Peruvian society. When distancing from class privilege, most respondents, like Ricardo (48, Established, Architect), opted to classify themselves from the derogative connotations of upper classness and placing themselves within the morally neutral category of ‘middle class’.

[If you had to self‐identify in terms of socioeconomic status or class, the home you grew up in and your current home, how would you identify them both?]

Very similar. But should I respond with a letter? ha, ha, ha

[However you prefer, any category]

(…) I don’t know. I would say middle class. (…) Middle, middle class. I don’t know. I don’t think I’m in the upper class at all. But a friend told me, ‘But if the middle class goes to César Vallejo^5^ when they leave their studies… how come you’re not upper class?’. Sure, it’s something… maybe it’s a flaw because you grow up with this idea that it’s almost bad to be in the upper class, right?

(Ricardo, 48, Established, Architect, Own emphasis)

These testimonies reflected a desire to appear ‘ordinary’ (Savage et al. 2001). By distancing themselves from these sectors, respondents reinforced a meritocratic narrative of the self, positioning themselves as separate from the privileges and arrogance associated with Peru's upper class (Montero‐Diaz 2019). These negative portrayals of the upper class are not limited to Lima or Peru, as evidenced by recent literature on class stereotypes (Piston 2018; Wu et al. 2018). Even if respondents may ‘implicitly’ favour the most privileged sectors in society, their attempts to discursively detach themselves from this sector are less dependent on their recognition of how distant or close are their material circumstances to those of the upper class than a morally charged distancing from this sector.

### 4.2 Affirming ‘Afro‐Peruvianness’, Distancing From ‘Whiteness’

Responses to questions about racial self‐identification offer a valuable opportunity to explore the frameworks and emotional aspects underlying respondents' experiences of racialisation. We can begin by examining the responses of those who identified as ‘Afro‐Peruvian’ or ‘Black’. Compared to the rest of the sample, the most notable aspect of their responses was their self‐assurance in their racial self‐identification. In some instances, like in Nora's (Long‐range, 64, University professor) testimony, identifying as ‘Afro‐Peruvian’ carried significant emotional weight. In her case, the ‘discovery’ of her racial roots was accompanied by her refusal to conform to the subtle yet powerful white standards of beauty, notably rejecting hair straightening.

I’m Afro‐Peruvian; that’s unavoidable. Plus, I feel proud to be so (…) Already at sixty, what does the whole world matter to me, right? I loosen my hair. I leave it long. I have never had it long. So, before, I always had my hair tied up because my head would look like that without it. So, they annoyed me because they called me ‘Black’, right? And it hurt, it really hurt

(Nora, Long‐range, 64, University professor)

Nora's testimony highlights how this racial identity combines distinct physical features associated with ‘Blackness’ (Benavides et al. 2006; Golash‐Boza 2010) and a shared awareness of the challenges Black individuals face in a deeply racist society (Valdivia 2014). A key part of this process involves recognising that some ‘Afro‐Peruvian’ individuals may partially adopt or even completely reject the cultural attributes tied to ‘Blackness’ (Meghji 2019). These varied responses are possible because of the existence of a relatively well‐defined cultural framework, with its own representatives and cultural signifiers, which individuals can incorporate into their identity. However, these aspects of the ‘Afro‐Peruvian’ experience seem to be exceptions rather than the norm in contemporary Peru's processes of racialisation.

In sharp contrast with the responses of ‘Afro‐Peruvian’ respondents were the only two individuals in the sample identifying as ‘White’. A notable aspect of their responses is their clear discomfort with the question. Moreover, their answers were tinged with a hint of embarrassment at identifying as ‘White’. Unlike respondents who confidently embraced an ‘Afro‐Peruvian’ identity, both only settled on this label after considering other options and comparing themselves to others. Their responses seemed more like admissions than proud affirmations of their identity.

[How do you identify yourself in ethnic‐racial terms?]

Wow, what a good question and how difficult. I’ve never thought about it. In most places… I am not lying… Well, I’ll give you some information, and you help me because I have doubts. I think I’m White. I mean, why am I saying this? Because outside of the work environment or a few other meetings, I’m usually the whitest person in the room. So, that happens to me a lot. So, I think my racial features are… Yes, I am, that is, White, I think… Me, my family too. I mean, my, my wife is whiter than me. She has green eyes. My son is… my son is a ghost… That allows me to understand [that] he is [White], but… I still confess that it is uncomfortable to talk about this.

(Fidel, Established, 38, Business executive, Own emphasis)

I am White, that is, White. Because it would be lying to you [to say something else]. I’m not Quechua. I’m not Awajún. I’m not… I come from a family of jungle settlers, who were all White, who invaded the jungle

(Raul, Long‐range, 54, Advisor of health policy)

But Fidel and Raul were hardly the only respondents whose testimonies manifested being interpellated by attributions to ‘whiteness’. The testimonies of most Established respondents in the sample were marked by similar hesitations and feelings regarding their ‘White’ status. However, they ultimately refrained from self‐identifying as ‘White’: some chose to self‐identify as ‘*Mestizo’* (5) while others opted not to give an answer (1). Interestingly, the majority of these respondents felt comfortable racially identifying their parents and other family members as ‘White’. Luciana's (Established, 45, Architect) testimony illustrates this pattern well. Since her husband was also one of the Established respondents in the sample,^6^ we can compare how they racially identified themselves and each other. Both grew up in similar privileged environments in Lima and pursued comparable academic and professional paths, and they similarly identified their family members as ‘White’. However, when it came to their own racial self‐identification, Luciana chose not to assign a racial label to herself—she is listed as ‘Don't Know’ in the racial self‐identification column in Table 1—while her husband identified as ‘*Mestizo’*. Nevertheless, when asked to label Luciana racially, he responded ‘White’.^7^

[How would you racially self‐identify?]

That is always a problem. I remember that they made us fill out these forms in the United States constantly. How you look… There were many options, and I didn’t know what I was. I have always answered, sometimes, I answered Mestizo, and then I realised that [I] was not so much [that category]. (…) It is assumed that between categories, I don’t know. Typically, they say that I am White. But I’m not as White as my sisters, for example, who are much whiter. I am the brunette in my family, but I don’t know, it’s difficult. I don’t know what to tell you. (…) Also, I think that in Peru, it’s a crazy thing. We all have an incredible mixture of blood inside, especially the entire population of the coast, right?’

(Luciana, Established, 45, Architect, Own emphasis)

[What category would you use to racially identify her?]

For my wife… [Yes], In the United States, we both responded Latinos, right? Now, in Lima, I would say she is White.

(Luciana’s husband)

Like Luciana, most Established respondents in the sample who rejected the ‘White’ label had an appearance that most Limeños would typically associate with it—most notably, having ‘clear skin’ (Kogan and Galarza 2017). These responses reflect attempts to distance themselves from whiteness as a way of downplaying privilege, mirroring the broader tendency among respondents to resist identification with the upper echelons of Lima's society. This resemblance is not coincidental; rather, it highlights the symbolic equivalence between whiteness and upper‐class status in Lima. This connection often leads individuals to reject these identities, regardless of how they are perceived by others (Montero‐Diaz 2019). As I will discuss, this strategy is facilitated by the widespread availability of the label ‘*Mestizo’*, which is accessible to virtually everyone.

### 4.3 ‘*Mestizo*’ Ambiguities

As shown in Table 1, ‘*Mestizo’* was the most frequently selected category for racial self‐identification. As observed earlier, for some Established respondents, choosing to identify as ‘*Mestizo’* served as a means of refraining from identifying as ‘White’. In these instances, their inclination to distance themselves from upper‐class status by opting for seemingly ‘neutral’ and ‘ordinary’ terms like ‘middle class’ mirrors their preference for identifying as ‘*Mestizo’* ^8^.

But what about upwardly mobile individuals who identify as ‘*Mestizo’*? Does this reflect a distancing from whiteness, as seen among Established respondents, or a form of racial affirmation, as among ‘Afro‐Peruvian’ participants? Around half of the upwardly mobile respondents, like Elena (Short‐range, 29, Sustainability Coordinator), identified as ‘*Mestizo’* without hesitation, often citing mixed ancestry—such as Quechua, Afro‐Peruvian, or Chinese—and naming regions outside Lima, like Ica, as part of their heritage. Others, like Leonardo (Long‐range, 39, University Professor), expressed similar confidence. His response reflects the moral legitimacy ‘*Mestizo’* holds for many, allowing them to sideline race in a context where everyone is seen as ‘*Mestizo’*. As in Leonardo's case, this is often tied to a rejection of race as a biological category and of the idea of ‘racial purity’.

Ah, I always say I am a Mestizo, that is, and I also directly have my grandfather who is Chinese, my father, who is from Ica, Ica, and my family who come from all over, so yes, I always identify myself as a Mestizo person.

(Elena, Short‐range, 29, Sustainability coordinator)

Damn, I’d say I’m like Homer Simpson. I simply don’t have colour. I don’t classify myself into colour, but if you force me to put it in biological terms, I am like all of Peru, which is Mestizo.

(Leonardo, Long‐range, 39, University professor)

While respondents like Elena and Leonardo confidently identified as ‘*Mestizo’*, about half of the upwardly mobile participants echoed the uncertainty and reluctance of Established respondents when it came to identifying as ‘White’. Despite the differing reasons behind their choice of ‘*Mestizo’*, all testimonies underscored the category's inherent ambiguity. Often, this aligned with arguments supporting colour blindness, either by denying the existence of race or endorsing ideas of *Mestizaje* (Moreno Figueroa 2022; Romero 2022). Unlike the relatively stable symbolic boundaries of ‘Afro‐Peruvian’ identity, ‘*Mestizo’* emerged as a catch‐all term—universal and readily accessible to those favouring a colour‐blind perspective.

### 4.4 From Whitening to Decolouring

While many upwardly mobile respondents considered that people who—like themselves—achieved a higher social status ‘whitened’ themselves, they also shared the idea that, no matter the efforts, there is a physical or *bodily* boundary preventing their full assimilation. Most respondents clearly identified individuals they considered ‘White’. They often provided detailed accounts of who they meant by ‘White’ people, ultimately emphasising physical appearance as the primary criterion for this label.

Jorge's (Long‐range, 31, Financial Analyst) account illustrates this point. He described his ‘White’ colleagues using several markers of high status in Lima, particularly those with significant symbolic weight, such as graduating from a prestigious school or having a ‘foreign’ surname (Reátegui et al. 2022). These markers carry strong class and racial connotations. In addition, Jorge underscored the physical appearance of his colleagues as ‘White’. For him, these two aspects of identity remain distinct, even though they overlap in cultural perceptions of whiteness. His testimony reveals that the cultural and social symbols of privilege highlight his colleagues' ‘White’ racial status, which ultimately depends on their physical characteristics. Furthermore, Jorge's critical remark at the end of his statement reflects the correlation between these elements: individuals with phenotypically ‘White’ traits enjoy all the social privileges associated with ‘whiteness’.

[Regarding social background, they came from quite privileged homes. If you had to think now in, let’s say, ethnic‐racial terms, how would you identify them?]

Whites. I mean, the truth is, they were White. There were surnames from abroad out there… let’s see, it’s the upper class, to put it in some way. In other words, although many of them had studied at La Pacifico [university]… more came because of recommendations: ‘Hey, look, he has studied at my school and so on.’ They all [were] very brilliant at their job, right? (…) There were one or two out there that maybe you would say, ‘This one isn’t that bright’. But the rest, yes, they were hardworking and everything. (…) But there was a clear marking and racial delimitation, which I don’t know if it was on purpose

(Jorge, Long‐range, 31, Financial analyst, Own emphasis)

Like Jorge, most testimonies underscored the importance of physical characteristics despite the cultural and embodied aspects of ‘whiteness’. Their testimonies manifested that as one ascends to the upper tiers of the class hierarchy, ‘colourism’ (Dixon 2019; Golash‐Boza 2010; Hunter 2007) remains a barrier that separates those perceived as ‘*Mestizo’* or ‘Afro‐Peruvian’ from those whose bodily features position them as ‘White’. This coloured boundary becomes increasingly salient as one moves to environments associated with the upper echelons of Lima's society, where non‐White individuals are increasingly more likely to experience racial stigmatisation and discrimination in environments dominated by ‘White’ people (Paredes 2007; Thorp and Paredes 2010).

The recognition of this insurmountable racialised boundary did not preclude upwardly mobile individuals from experiencing shifts in their racial status following social mobility. Lucia's (Long‐range, 39, Lawyer in a firm) reflections on the prospects of ‘whitening’ in Peru illustrate this dynamic. Drawing from her own experiences, Lucia acknowledged a notable difference in how she is currently racially perceived compared to her parents. She recognised that, despite sharing similar physical characteristics with her father, they are not positioned in the same way. While her father would likely encounter discrimination due to his social background and trajectory, Lucia believed that she would be less likely to face such treatment because of her current status, evident in her knowledge, appearance, and education. The latter plays a significant role in her life story, framing it as the decisive factor enabling her to attain a higher status. However, Lucia framed education not only as a means for social mobility but also as a form of ‘miscegenation’. Rather than offering the chance to adopt a ‘White’ status, for Lucia, education and its impact on her trajectory has counterbalanced the risk of being *reduced* as someone's ‘*Cholo’*, providing greater opportunities for respect (e.g., ‘it has allowed me to assert my rights a bit’).

[There is a widespread idea that ‘money whitens’. Do you think that can apply here in Peru?]

Education? I think so. Education is like a Mestizaje (miscenegation). Before, it was well… My dad is a person who comes from the provinces, and he came washing dishes to work washing dishes when he arrived. He hadn’t even finished school; he had to finish it while working. But he was… Well, what they would call a Cholo here, a cholo you can cholear. On the other hand, I feel that, despite the fact that I am racially very similar to him, they can’t cholear me. I’ve become mestiz… mestizised? I don’t know how to say it. Because of education. Because I know how to talk, I know how to behave, I know my rights, I know some things. I don’t know if I’ve whitened, I don’t know if that term… maybe yes, maybe that’s the correct term, but I don’t feel whiter. I like my skin colour; I do not deny my origins. But it has allowed me to enforce my rights a bit.

(Lucia, Long‐range, 39, Lawyer in a firm, Own emphasis)

Most upwardly mobile respondents echoed Lucia's views on the feasibility of ‘whitening’ in Peru. They highlighted that, rather than merely ‘passing’ as ‘White’ (Brubaker 2016), a ‘successful career’ allowed them to gain respect and avoid being labelled as ‘*Cholo’*. While ‘*Mestizo’* appeared as a seemingly ‘neutral’ and ambiguous label, its synonymous ‘*Cholo’* revealed in testimonies their continual lived experiences of racial stigma and discrimination. As the below testimonies of Ernesto and Cristian show, adoption of specific ways of dressing and speaking emerged as strategies for countering discrimination and stigma faced by non‐White individuals trying to navigate the ‘Whiteworld’ (Rollock et al. 2015). Rather than simply seeking ‘White’ status, these practices represent attempts to minimise the likelihood of experiencing discrimination, that is, ‘*choleo’*, in elite settings.

[The security at the door told me] ‘No, it’s not full’. And they let other people in, but not me. I don’t know how it happened. (…) but I showed that I was from the Catholic University, and they let me in

(Ernesto, Long‐range, 54, Finance manager)

I was coming home from a party, and I think I had put on a beanie because I had short hair, and a municipal guard stopped me half a block from my house. And he told me, ‘We thought you were stealing cars’. [He Said] such a thing. For me, it was super harsh. I thought, ‘Clearly there is a super strong racial profiling here’.

(Cristian, Short‐range, 28, Journalist)

I argue that the experiences of upwardly mobile ‘*Mestizo’* individuals assimilating into elite circles are framed as instances of what I term ‘decolouring’. This process involves avoiding derogatory racial and class positions, such as being labelled a ‘*Cholo’*, by adopting characteristics associated with ‘whiteness’ to mitigate discrimination in environments defined by colourism (Dixon 2019; Hunter 2007). Unlike the relatively stable boundaries of ‘Afro‐Peruvian’ identity, the ‘*Mestizo’* category is marked by ambiguity, often serving as a vague, inclusive concept that anyone can adopt. This prevents it from being a distinct non‐White identity that could challenge the association between ‘whiteness’ and ‘upper classness’. In contrast to ‘de‐Indianisation’ in the Andes, which focuses on cultural processes, decolouring highlights the importance of racialised bodily distinctions and guards against the assumption that ‘Mestizo’ individuals simply become ‘White’ as they move up the social ladder, given the colour divide separating non‐White individuals from their White counterparts.

While also involving a distancing from a stigmatised racial condition, the notion of decolouring among ‘*Mestizos’* in the sample differs in important ways from Bonilla‐Silva's (2002) ‘honorary White’ thesis and De la Cadena's (1998) account of ‘de‐Indianisation’. Unlike ‘light‐skinned Latinos’ or ‘Indios’, who can engage in identity work by drawing on their ethnic backgrounds, they lack a clearly defined racial identity to assert. In contrast to ‘Afro‐Peruvians’, who navigate representations of ‘Black’ and ‘White’ cultures, ‘*Mestizo’* identities are marked by universality, reflected in statements such as: ‘I'm like all Peruvians, Mestizo’ (Leonardo, Long‐range, 39, university professor). The ambiguity of ‘*Mestizaje’* and its connections to class inequalities provide the context for decolouring, where upwardly mobile ‘*Mestizos’* face not only the challenges of achieving higher class status but also shifts in how they are racially positioned. Rather than ‘whitening’, mobility thus defuses the risk of being relegated to the derogatory status of ‘*Cholo’*.

## 5 Conclusion

In this article, I have explored how mobility shapes the identification practices of upwardly mobile individuals within Lima's dominant class, focussing on the intersections of class and race. I analysed respondents' reasoning and emotional responses, as well as the symbolic connections between class and racial identities. Respondents' narratives highlight the cultural significance and specificity of the ‘Afro‐Peruvian’ identity, which emerges as a meaningful social condition that transcends class. This contrasts sharply with the associations tied to ‘*Mestizo’* and ‘White’ identities. While respondents view ‘*Mestizo’* as neutral and linked to the middle class, they associate ‘White’ identities with privilege and the upper class. Consequently, most respondents avoid identifying as ‘White’ or upper‐class, preferring the less contentious labels of ‘*Mestizo’* and middle‐class, regardless of how others perceive them.

I also examined how mobility shapes status changes for ‘*Mestizo’* and ‘Afro‐Peruvian’ respondents. For ‘Afro‐Peruvians’, navigating elite spaces often requires negotiating their identity within the ‘WhiteWorld’ (Rollock et al. 2015). Despite these challenges, their racial identity remains distinct and central to their self‐conception (Golash‐Boza 2010).

In contrast, upwardly mobile ‘Mestizos’ described how mobility reduces the likelihood of being racialised as ‘*Cholos’*—a derogatory and racialised term. However, this shift does not equate to their assimilation into ‘White’‐dominated spaces. Respondents reported both subtle and overt forms of segregation, particularly within the ‘whitest’ elite strata, highlighting the persistent role of ‘colourism’ in Peru's class system.

To capture the specific experiences of upwardly mobile ‘Mestizos’, I introduced the concept of ‘decolouring’. This process involves adopting traits associated with ‘whiteness’ and class privilege, which reduces the chances of being labelled ‘*Cholo’* but does not grant full ‘White’ or ‘honorary White’ status. Instead, it creates uncertainty about their social position. Unlike the ‘Afro‐Peruvian’ identity, ‘*Mestizo’* lacks clear cultural markers, allowing individuals to use it strategically to downplay privilege. However, many upwardly mobile ‘*Mestizos’* experience ‘decolouring’ as a precarious and ongoing process, where elite interactions remain overshadowed by the risk of being racialised as ‘*Cholo’*.

Overall, this article contributes to the study of racialisation processes and their connection to social mobility. In particular, it offers an interpretation that may illuminate racialised dynamics in other contexts characterised by porous racial boundaries and the enduring dominance of ‘whiteness’.

## Ethics Statement

The study from which the data of this article was generated was approved by the Social Sciences School Panel of the University of Manchester on the 16th of May of 2022 (Ref: 2022‐14239‐23511).

## Conflicts of Interest

The author declares no conflicts of interest.

## Data Availability

The research data are not shared because participants did not provide written consent for public disclosure.

## Endnotes

^1^
On average, the occupations linked to the ‘dominant class’ show a wide variation in levels of economic capital. However, even those occupations within this class that have the lowest economic capital—but the highest cultural capital, according to the class scheme—still earn more than those in other classes: 28% more than the ‘intermediate class’, 57% more than the ‘working class’, and 80% more than ‘rural workers’.

^2^
I based their mobility status on the occupation held by the parent within the highest class.

^3^
Only one Established respondent mentioned that one of his grandmothers had attended university.

^4^
See Table 1 for the full list of respondents' identification across the axes of race and class.

^5^
The César Vallejo University is a recently created private university with its headquarters in the North region of La Libertad in Peru.

^6^
Luciana and her husband were invited to participate through the institutional email addresses of the universities where they work, without the researcher being aware of their marital status. It was only after Luciana agreed to be interviewed that she informed the researcher about her husband's involvement in the study.

^7^
Luciana did not respond to her husband's racial identification because she assumed that he already provided it during our previous interview.
